# Solution of India-Rubber in Local Treatment of Psoriasis

**Published:** 1876-12

**Authors:** 


Solution of India-Rubber in Local Treatment of Psoriasis. Mr. Wyndham Cottle has found this material useful in chronic cases of psoriasis, where there is an excessive formation of dry scales, especially in the neighborhood of the joints, where the scales form crusts, are hard, and render the movement of the part painful. The crusts and scales being removed and the absence of grease insured by wiping the parts with ether, and the skin dried, a solution of the india-rubber is applied with a brush and the application renewed as often as is needful to maintain a contiuous covering over the affected skin. He recommends a solution composed of india-rubber half an ounce, and chloroform eleven and a half ounces. He has met with more rapid recovery in these cases by this application than by the ordinary local measures, from the fact that these coverings prevent evaporation and hinder the loss of the natural perspiration of the part. He thinks the same treatment is applicable to some conditions of chronic eczema.—*The Lancet.*

